# β-Glucosidase *VmGlu2* Contributes to the Virulence of *Valsa mali* in Apple Tree

**DOI:** 10.3389/fmicb.2021.695112

**Published:** 2021-07-30

**Authors:** Yan Huang, Chunlei Yu, Cuicui Sun, Muhammad Saleem, Pingliang Li, Baohua Li, Caixia Wang

**Affiliations:** ^1^Key Laboratory of Integrated Crop Pest Management of Shandong Province, Shandong Province Key Laboratory of Applied Mycology, College of Plant Health and Medicine, Qingdao Agricultural University, Qingdao, China; ^2^Department of Biological Sciences, Alabama State University, Montgomery, AL, United States

**Keywords:** *Valsa mali*, β-glucosidase activity, toxin level, pathogenicity, apple tree

## Abstract

The apple tree canker is caused by *Valsa mali*, which produces major pathogenic factors involving multiple cell wall-degrading enzymes (CWDEs) and toxins. The β-glucosidases are among the main CWDEs, and thus, they play important roles in the virulence of necrotrophic pathogens. However, the specific roles of β-glucosidases in the virulence of *V. mlai* remain largely unknown. In this study, we identified a β-glucosidase gene, V*mGlu2*, which was upregulated during the *V. mali* infection. We found that VmGlu2 protein had high enzyme activity of β-glucosidase using p-nitrophenyl-β-D-glucopyranoside (pNPG) as a substrate, while the VmGlu2 could convert phloridzin to phloretin with the release of glucose. The deletion and overexpression of *VmGlu2* showed no effect on vegetative growth, but gene deletion mutants of *V. mlai* showed significantly reduced pycnidia formation. The gene deletion mutants had lower β-glucosidase activities and toxin levels as compared to the wild-type strain. Therefore, these mutants showed a reduced virulence. Moreover, the overexpression of *VmGlu2* did not affect toxin levels, but it significantly enhanced β-glucosidase activities, which resulted in an increased pathogenicity. Thus, we conclude that *VmGlu2* is required for the full virulence of *V. mali*. These results provide valuable evidence to the complex role of CWDEs in the fungal pathogenicity.

## Introduction

The hydrolytic enzymes of pathogens are important factors in pathogenesis because they determine the accumulation of secondary metabolites and degradation of host plant tissues (Morrissey and Osbourn, 1999; Schirawski et al., 2006; Kubicek et al., 2014). The carbohydrate active enzymes degrade cell wall polymers cell wall-degrading enzymes (CWDEs), and facilitate pathogens to invade plant tissues for nutrient acquisition (Hématy et al., 2009; Zhao et al., 2013). These enzymes that regulate the breakdown of cellulose, xylan, and pectin, are particularly important for phytopathogenic fungi, because they lack specialized penetration structures (Kikot et al., 2009; Gibson et al., 2011). There are evidences that the disruption or modification of genes encoding CWDEs may reflect their direct involvement in the infection and disease (Zhang et al., 2009; Yu et al., 2018). For example, the deletion of xylanase genes *Xyn11A* and *BcXyl1* had a marked effect on the ability of *Botrytis cinerea* to infect host plants (Brito et al., 2006; Yang et al., 2018), while the gene deletion mutant of *VdCUT11*, encoding a cutinase, impaired the virulence of *Verticillium dahliae* (Gui et al., 2018). In *Phytophthora sojae*, the deletion and overexpression of xyloglucanase gene *PsXEG1* severely reduced its virulence (Ma et al., 2015). However, the specific roles of the majority of CWDEs in the virulence remain largely unknown.

The β-glucosidases (EC 3.3.1.21) play an important role in the degradation of the cellulose that catalyze the hydrolysis of glucosides and oligosaccharides by releasing glucose (Kubicek et al., 2014). β-glucosidases are predominantly found in the glycoside hydrolases 1 (GH1) and GH3 families. Both GH families hydrolyze their respective target substrates with a net retention of the configuration of the anomeric carbon (Collins et al., 2007; Tiwari et al., 2013). These enzymes have distinct biological roles, and thus have been well characterized for their wide applications in the biomedical industry (Tokpohozin et al., 2016; Salgado et al., 2018). In addition, these enzymes also play a vital role in the production of different energy sources (biofuel) during biomass conversion (Bayer et al., 2007).

Nevertheless, the specific roles of β-glucosidases in virulence are reported in several pathogens. In *B. cinerea*, the positive correlation was found between the β-glucosidase activities and the pathogenicity (Sasaki and Nagayama, 1994, 1996), while the deletion of *BcSUN1*, encoding a β-glucosidase, had a marked effect on the production of reproductive structures as well as the ability of the pathogen to infect bean, tomato and tobacco plants (Pérez-Hernández et al., 2017). Jourdan et al. (2018) demonstrated that β-glucosidase *BglC* plays an important role in the virulence of *Streptomyces scabies*, by affecting the intracellular accumulation of signals that trigger the thaxtomin A biosynthesis. Thaxtomin is the main phytotoxin produced by *S. scabies*. However, the underlying mechanism of *BglC* in the virulence of *S. scabies* is more complex than commonly perceived (Jourdan et al., 2018).

*Valsa mali*, a typical necrotrophic fungus, is the causative agent of *Valsa* canker on apple tree via the production of toxic compounds (phytotoxins) and CWDEs (Chen et al., 2012; Wang et al., 2014). A whole-genome analysis also revealed that *V. mali* contained a number of genes associated with the hydrolytic enzymes and secondary metabolite biosynthesis (Yin et al., 2015). Transcriptome profiling had also suggested that cell wall degradation is important for the infection of apple tree by the *V. mali* (Ke et al., 2014). Some studies investigated the role of CWDEs such as xylanase, polygalacturonases and ferulic acid esterases in the virulence of *V. mali* (Xu et al., 2016, 2018; Yu et al., 2018). Previous studies have found that *V. mali* secretes β-glucosidases with high activity, and its encoding genes are up-regulated during the pathogen infection (Chen et al., 2012; Li et al., 2017). This indicates that β-glucosidases play important roles in the virulence of this organism. In addition, the toxic compounds produced by *V. mali* are the degradation products of phloridzin, and the first step in the degradation process involves the conversion of phloridzin into phloretin with the release of glucose (Natsume et al., 1982). Gaucher et al. (2013) demonstrated that high β-glucosidase activity in the apple tree infected by *Erwinia amylovora* were responsible for the conversion of phloridzin into phloretin accompanied by the release of large amounts of glucose that stimulated pathogen growth in the diseased tissues. However, we know little about the comprehensive role of β-glucosidase in the virulence of *V. mali*, and thus it requires immediate research efforts.

Here, we identified a gene, *VmGlu2*, from *V. mali*, which is up-regulated in apple tree during the pathogen infection. The VmGlu2 is a member of GH1 with β-glucosidase activity when p-nitrophenyl-β-D-glucopyranoside (pNPG) or phloridzin as a substrate. Moreover, *VmGlu2* is required for full pathogenicity of *V. mali* in apple tree, and involved in pycnidia formation. Our results indicate that VmGlu2 has a major role in the virulence of *V. mali* and provides important information for us to understand the pathogenicity of necrotrophic fungus.

## Materials and Methods

### Strains and Culture Conditions

The *V. mali* wild-type strain LXS080601 was grown on the potato dextrose agar (PDA, 200 g of potato, 20 g of dextrose, and 15 g of agar per liter) at 25°C in the dark. The gene deletion and complemented strains were cultured on PDA supplemented with 100 mg/mL of hygromycin B or geneticin G418 (Sigma, St. Louis, MO, United States). The *Escherichia coli* strains were grown in the lysogeny broth (LB) with appropriate antibiotics at 37°C. The measured colony diameter was used to calculate the growth rate of different strains on PDA medium.

### Identification of *VmGlu2* in *V. mali* and Sequence Analysis

The genomic DNA and total RNA were extracted from the *V. mali* mycelium. The cDNA was synthesized using the Prime Script RT reagent Kit with gDNA Eraser (TaKaRa, Dalian, China) using an oligo (dT)_1__2__–__1__8_ primer. The gene *VmGlu2*, with putative β-glucosidase activity and high transcript levels during *V. mali* infection was cloned. Primer pairs for the cloning of *VmGlu2* were synthesized by TsingKe (Beijing, China) (Supplementary Table 1). The PCRs were performed with PrimerSTAR Max DNA polymerase and cloned to T-Vector pMD 19 Simple (TaKaRa, Dalian, China), according to the manufacturer’s instructions.

The amino acid sequences of β-glucosidase from other strains in this study were obtained from the NCBI GenBank. All the homology searches were carried out on the NCBI BLAST server. The obtained sequences were compared with the sequences from *V. mali* (KX013493). Maximum likelihood (ML) method, as implemented in MEGA7, was used to infer the phylogenetic tree with 1000 bootstrapping replicates. Multiple sequence alignments of *VmGlu2*, and other well characterized β-glucosidase genes were performed using the DNAMAN (version 6.0) with all the parameters set at the default values.

### Expression and Purification of Recombinant Protein

The cDNA fragment of *VmGlu2* was subcloned into the pET-32a vector by homologous recombination using the ClonExpress II One Step Cloning Kit (Vazyme, Nanjing, China). The vector construct was transformed into *E. coli* strain Rosetta while the soluble recombinant protein of VmGlu2 was obtained after induction with 0.5 mM isopropyl β-D-thiogalactopyranoside (IPTG) for 16 h at 15° (Shi et al., 2015). The purification of recombinant VmGlu2 protein from the culture was performed using Ni-NTA Spin Column (Qiagen, Beijing, China). The expression and purification of recombinant protein were detected by the sodium dodecyl sulfate polyacrylamide gel electrophoresis (SDS-PAGE) and Western blot.

### Assays for β-Glucosidase Activities

The β-glucosidase activities were determined using pNPG as a substrate according to a previously established method (Alconada and Martínez, 1996). The enzyme activity was measured at 50° in 50 mmol/L sodium-citrate buffer with a pH of 5.5. One unit of enzyme activity was defined as the amount of product (μmol) formed per min under the assay conditions.

The activities of β-glucosidase were also measured using the phloridzin as a substrate by high performance liquid chromatography (HPLC) according to Gaucher et al. (2013) with some modifications. Activity was assayed in 1 mL of reaction mixture containing 0.5 mmol/L phloridzin and about 20 μg purified recombinant protein in 50 mmol/L phosphate buffer (pH 7.0) at 25° under continuous stirring conditions. The samples were analyzed by HPLC on an Agilent 1200 (Agilent Technologies, Santa Clara, United States), and separated on a C18 column (250 mm × 4.6 mm, 5 μm) at 30°, with a detection wavelength at 285 nm.

### Detection of Gene Expression by qRT-PCR

The mycelia plugs of *V. mali* were used to inoculate apple twigs described by Yu et al. (2018). The lesion border of apple bark tissues was sampled at different time points (0, 6, 12, 24, 48, 72, and 120 hpi). For samples at 0 hpi, bark tissues around inoculation sites containing mycelium plugs were collected. The RNA was extract from bark tissues using the RNAiso Plus Kit (TaKaRa, Dalian, China), and then the cDNA was synthesized with a Reverse Transcription Kit. All of the qRT-PCR experiments were conducted in a LightCycler 480II PCR Detection System (Roche, Mannheim, Germany) with SYBR Master Mix (TaKaRa, Dalian, China) following the manufacturer’s protocol. The *EF1-a* of *V. mali* was used as an internal control, while primers are given (Supplementary Table 1). The relative expression level of *VmGlu2* was calculated using the 2^–ΔΔ*CT*^ method (Livak and Schmittgen, 2001). Data from three biological replicates were used to calculate the means and standard deviation. The whole experiment were repeated twice.

### Generation of *VmGlu2* Deletion and Complementary Mutants

To obtain the *VmGlu2* deletion mutants, the polyethylene glycol (PEG)-mediated homologous recombination was performed as described previously (Yu et al., 2018). *VmGlu2* and about 1200 bp flanking sequences of the gene were amplified from the genomic DNA of the wild-type strain LXS080601. The hygromycin B phosphotransferase gene (*HPH*) gene was amplified with primers HPH-F and HPH-R from the vector pBS (Supplementary Table 1). Two flanking fragments and *HPH* resistance cassette were constructed into a fusion fragment (3,383 bp) using a nested PCR reaction. The fusion fragment was later transformed into the protoplasts of *V. mali* strain LXS080601, and the transformants were screened by culturing on medium with 100 μg/mL of hygromycin (Yu et al., 2018). More than 600 transformants were detected by PCR using the detection primer pairs *VmGlu2*-I-F/R. The putative gene deletion mutants were then validated by PCR using four primer pairs (Supplementary Table 1). The gene deletion mutants were finally verified by Southern hybridization with the DIG DNA Labeling and Detection Kit II (Roche, Mannheim, Germany) following the instruction manual.

To generate a complemented strain, the entire *VmGlu2* gene with upstream fragment was amplified from genomic DNA using the primer pair 1491-CM-F/R (Supplementary Table 1). The PCR products were cloned into the plasmid pYF11 using yeast gap repair and then verified by the sequencing analysis. The recombinant vector pYF11-*Glu2* was then transformed into the gene deletion mutant via PEG-mediated method. The transformants were selected using G418, and confirmed by PCR with the corresponding primers (Supplementary Table 1).

### Generation of *VmGlu2* Overexpression Transformants

The full-length fragment of *VmGlu2* was amplified from the genomic DNA of *V. mali* and then cloned into plasmid pYF11 using the ClonExpress II One Step Cloning Kit (Vazyme, Nanjing, China). The construct was verified by sequencing and then transformed into the wild-type strain LXS080601 as described above. Transformants were screened by PCR with corresponding primers outside the cloning sites of the pYF11. Relative transcript levels of *VmGlu2* in mycelia grown on PDA and inoculated apple twigs were determined as described above. Transformants were further verified by Western blot with Anti-GFP antibody (Abcam, United Kingdom).

### Vegetative Growth, Pycnidia Formation, and Pathogenicity Assays

The vegetative growth of *VmGlu2* deletion mutants and overexpression transformants was determined as described by Yu et al. (2018). Colony shape and color were observed, and then the colony diameters were measured. For determination of the dry weight of mycelia, plugs were inoculated into PDB media (potato dextrose broth) at 150 rpm and 25° for 7 days. For pycnidia formation, all the strains were grown on PDA under UV-light (365 nm) at 25° for 4 weeks. The assays were carried out three times and each experiment with four replicates.

For pathogenicity assays, we selected healthy apple leaves and 1-year old twigs (*Malus domestica* Borkh. cv. “Fuji”), which were sterilized with 75% ethanol, and spaced wounds were made as described by Yu et al. (2018). The mycelia plugs of all strains were used to inoculate the wounds. The inoculated leaves and twigs were placed in trays to maintain humidity at 25° in the dark. Then, the lesion length was measured and the photography was performed at different time intervals. The assays were repeated three times, with at least 10 leaves and twigs per treatment.

### Determination of Toxins Production

For toxins production, the mycelium plugs of different strains were inoculated into apple branch extract media. After culturing for 7 days at 150 rpm/min and 25°C with a 12-h photoperiod, the supernatant was collected. The liquid media inoculated with PDA was used as a control. Toxins were extracted and detected by HPLC according to the method described by Wang et al. (2014). Each experiment was done in triplicate.

### Statistical Analysis

All statistical analysis was conducted using SPSS software (Version 19.0, SPSS Inc., Shanghai, China). All the data collected were subjected to analysis of variance (ANOVA) followed by Duncan’s multiple range tests. The asterisks indicate a statistically significant difference with the wild-type strain (*P* < 0.05).

## Results

### Sequence Identification and Analysis of *VmGlu2*

**FIGURE 1:**
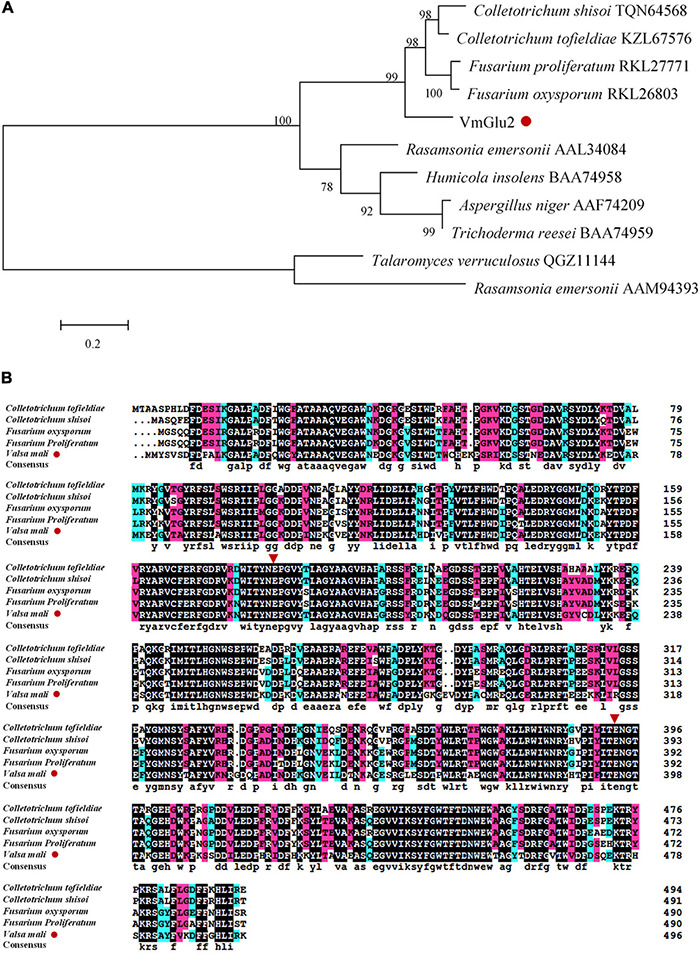
Bioinformatics analysis of VmGlu2 (Accession number: KX013493). **(A)** Phylogenetic analysis of VmGlu2 with the well-characterized β-glucosidase from GH1 family. The phylogram was generated by the Maximum likelihood method implemented in the MEGA7. Numbers beside each node indicate bootstrap values as a percentage of 1,000 replicates. Species names are followed by the accession numbers of β-glucosidase protein. **(B)** Multiple alignments of the amino acid sequences of VmGlu2 with known β-glucosidases from other fungi are given. Red inverted triangles indicate the catalytic residues among GH1 enzymes.

The *VmGlu2* gene was amplified by PCR using cDNA of *V. mali* as template, and then confirmed by the sequencing (Supplementary Figure 1A). The full-length cDNA of VmGlu2 is 1,677 bp containing 5°- and 3°-non-coding regions, as well an open reading frame of 1,491 bp that encodes a protein with a calculated molecular mass of 54.6 kDa. The DNA fragment was also 1,491 bp, with no intron. Using the Signal P5.0 server, VmGlu2 was predicted that no signal peptide is present. NCBI’s conserved domain database showed that VmGlu2 had a β-glucosidase (Bg1B) conserved domain, which belonged to a GH1 family (Supplementary Figure 1B). The sequence comparison demonstrated that VmGlu2 exhibited high homology with the well-characterized β-glucosidase. A phylogenetic tree was constructed with β-glucosidase from other strains (Figure 1A). The sequence alignments of VmGlu2 with four characterized GH1 β-glucosidases showed that VmGlu2 had the catalytic residues among these enzymes that are related to their activity, in which Glu395 acts as a nucleophile and the Glu181 acts as an acid/base (de Giuseppe et al., 2014). The sequence alignment is shown (Figure 1B).

### Confirming the β-Glucosidase Activity of VmGlu2 Protein

**FIGURE 2:**
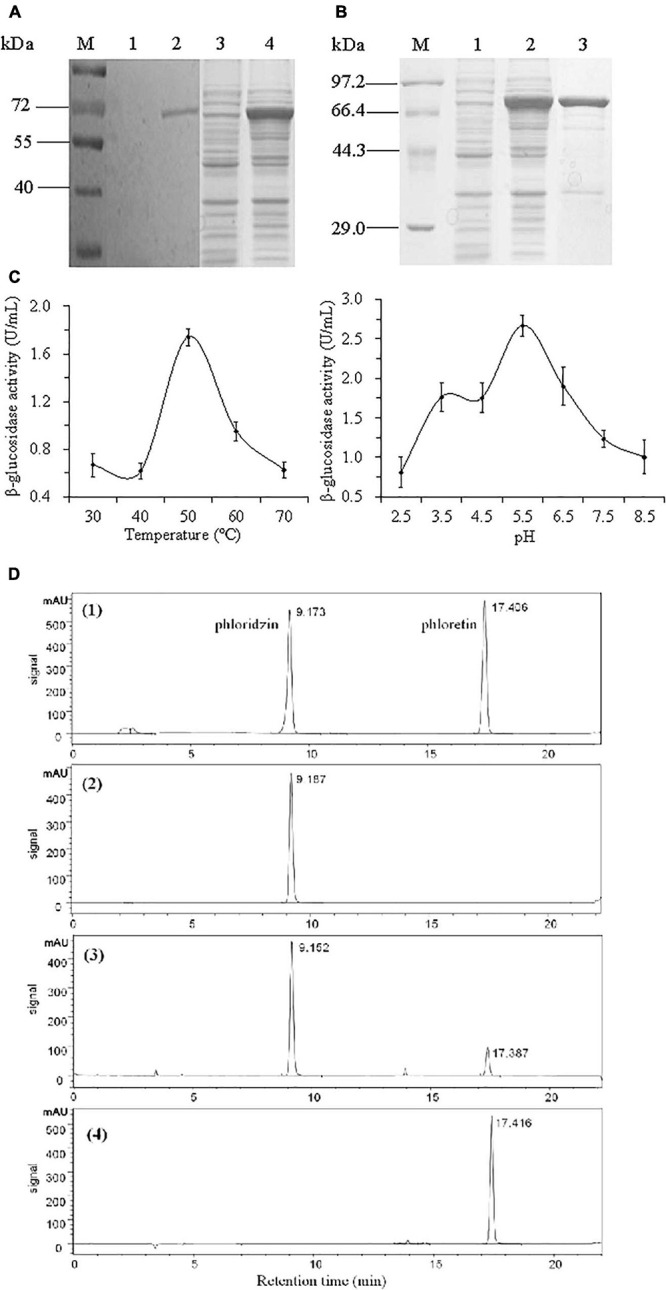
The determination of VmGlu2 recombinant protein expression, purification and enzyme activities. **(A)** Detection of the expression of VmHbh2 recombinant protein by Western blot. Lane M, protein molecular weight marker (Low); Lane 1 and 3, the empty vector as control (about 20 μg of total protein); Lane 2 and 4, the supernatant of the induced cells at 15 ° (about 30 μg of total protein). **(B)** Detection of the purified VmHbh2 recombinant protein by SDS-PAGE. Lane M, protein molecular weight marker (High); Lane1, the empty vector as control; Lane 2, the supernatant of the induced cells at 15 °, Lane 3, the purified recombinant protein. **(C)** Enzymatic activity of VmGlu2 recombinant protein when using pNPG as a substrate. **(D)** Enzymatic activity of VmGlu2 recombinant protein when using phloridzin as a substrate, and the data show the conversion of phloridzin into phloretin as determined by HPLC. (1), the standard of phloridzin and phloretin; (2), VmGlu2 recombinant protein and phloridzin (1 mg/mL) incubation for 0 h; (3), VmGlu2 recombinant protein and phloridzin (0.91 mg/mL) incubation for 24 h; (4), VmGlu2 recombinant protein and phloridzin (not detected) incubation for 72 h. The data next to the peak are the retention times for each compound.

In order to determine the enzymatic activity of Bg1B, the *VmGlu2* was cloned into pET-32a for heterologous expression in *E. coli* with a six histidine tag fused to the N-terminus part. The VmGlu2 recombinant protein with the expected molecular weight (MW) was obtained and verified by Western blot with Anti-His antibody (Figure 2A). We got the purified recombinant protein through Ni-nitrilotriacetic acid (Ni-NTA) Spin Column, and checked the protein by SDS-PAGE (Figure 2B).

The β-glucosidase activity of VmGlu2 protein was measured using pNPG as substrate at different temperatures (from 30 to 70°C) and pH values (pH 2.5–7.5). The enzyme activity remained constant from 30 to 40°C, dramatically increased to the highest value of 1.74 U/mL at 50°C, then declined abruptly to 36% of the maximal activity at 70°C. The optimal pH of VmGlu2 is around pH 5.5 as the enzyme maintained high activity between pH 3.5 and pH 6.5, while its activity declined rapidly to 30 and 37% of its optimum at pH 2.5 and pH 8.5, respectively (Figure 2C). In addition, we determined its activity when phloridzin as substrate by high performance liquid chromatography (HPLC). The Figure 2D showed that VmGlu2 had high activity to convert phloridzin into phloretin, with 78 and 100% conversion rate at 24 and 72 h, respectively.

### Expression Profile of VmGlu2

**FIGURE 3:**
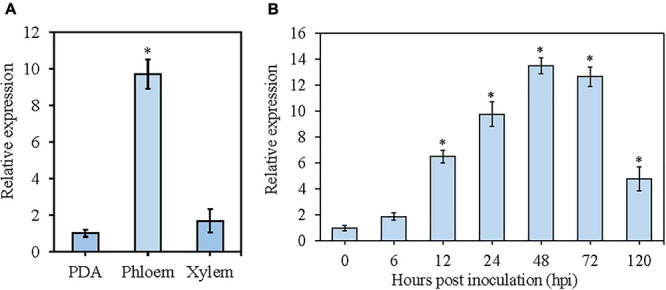
The expression levels of *VmGlu2* in different apple tissues at time points post-inoculation determined by qRT-PCR. The expression level of *V. mali EF1-a* gene was used as an endogenous control, and the means and standard deviation of the relative expression levels were calculated from three independent biological replicates. **(A)** Relative expression levels of *VmGlu2* in different apple tissues at 72 hpi. The expression level of *VmGlu2* in the mycelia grown on PDA was normalized to one. Asterisks represent significant differences (*P* < 0.05) in expression levels relative to that in PDA. **(B)** Relative expression levels of *VmGlu2* at 0, 6, 12, 24, 48, 72, and 120 hpi. Asterisks represent significant differences (*P* < 0.05) in expression levels as compared to that at 0 hpi.

To examine the expression level of *VmGlu2* in different apple tissues, we sampled infected apple phloem and xylem tissues at 72 h post inoculation (hpi) and compared the expression level with that of mycelia grown on PDA. qRT-PCR analysis showed that the expression level of *VmGlu2* were significantly up-regulated in the apple phloem (9.7-fold change) as compared to its expression in the mycelia grown on PDA. During the infection stage, the up-regulation trend of *VmGlu2* in apple xylem (1.7-fold change) had no significant difference relative to its expression levels in PDA (Figure 3A).

We also determined the expression level of *VmGlu2* during the interaction of *V. mali* with apple twigs (Figure 3B). To do this, we sampled infected apple phloem tissues at seven different time points (0, 6, 12, 24, 48, 72, and 120 hpi), and then compared gene expression levels to those of mycelia grown on PDA. The transcript level of *VmGlu2* was significantly up-regulated from 12 hpi, gradually increased and reached the highest with a 13.5-fold change at 48 hpi, and then declined sharply from 72 to 120 hpi (4.8-fold change). Overall, the high induction of *VmGlu2* during infection suggests its potential role in the pathogenicity of *V. mali*.

### *VmGlu2* Is Not Necessary for the Vegetative Growth but Pycnidia Formation of *V. mali*

To verify the functional role of *VmGlu2* in *V. mali*, we successfully knocked out the gene by PEG-mediated protoplasts transformation method. The mutants were examined via PCR assays and confirmed by Southern blot (Supplementary Figure 2). When probed with *HPH* fragment, the wild-type (WT) sample showed no hybridization signal. However, a clear hybridization signal of the predicted size was appeared on analysis of the gene deletion mutants. For the complementation of *VmGlu2* deletion mutant, a complementary construct was generated and transformed into the gene deletion mutant. All the complementation transformants were confirmed by PCR assays (Supplementary Figure 3A). In addition, RT-PCR analysis were also performed to confirm that the target *VmGlu2* was knocked out in the gene deletion mutants and that the complementation strains contained the target gene (Supplementary Figure 3B).

**FIGURE 4:**
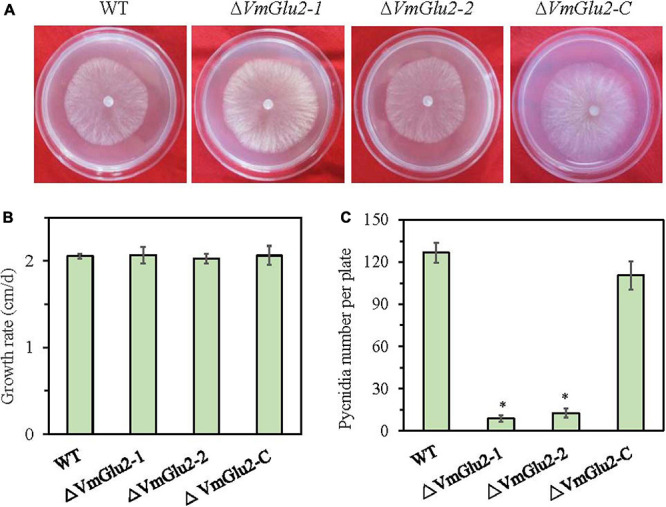
Colony morphology, growth and development of the wild-type stain (WT), *VmGlu2* deletion mutants (Δ*VmGlu2*) and complementary strain (Δ*VmGlu2-C*). **(A)** The colony phenotype of different strains growing on PDA at 25° in the dark for 2 days. **(B)** The mycelia growth rate of different strains on PDA at 25° for 3 days. **(C)** Number of pycnidia produced on per 9.0 cm petri plate under UV-light (365 nm) at 25°. Bars indicate standard deviation of means of four replicates. Asterisks represent significant differences (*P* < 0.05) in the gene deletion mutants as compared to that in the WT.

To determine whether *VmHbhs* plays a role in *V. mali* growth and development, we investigated the colony, growth rate, and pycnidia formation of the *VmGlu2* deletion mutants and the wild-type strain. However, no obvious differences either in colony morphology or growth rate were observed between the *VmGlu2* deletion mutants and the wild-type strain (Figures 4A,B). Assessment of the pycnidia formation by Δ*VmGlu2* was performed on PDA under UV-light (365 nm) at 25°. The *VmGlu2* deletion mutants produced less than 13 pycnidia per plate, while the wild-type strain produced more than 126 pycnidia per plate. Furthermore, the complementation strain Δ*VmGlu2-C* by reintroducing *VmGlu2* restored the wild-type strain pycnidia formation (Figure 4C).

### Deletion of *VmGlu2* Reduced the Virulence of *V. mali*

**FIGURE 5:**
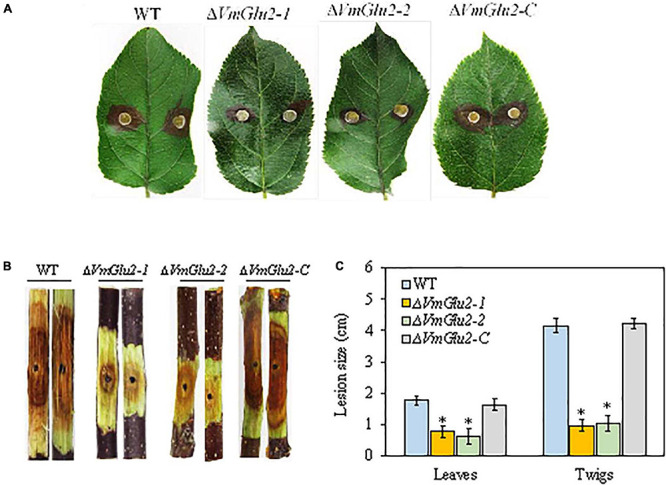
Pathogenicity tests of the WT, *VmGlu2* deletion, and complementation mutants on the leaves and twigs of *M. domestica* Borkh. cv. Fuji. **(A)** The infected phenotype of apple leaves inoculated with different strains at 3 dpi. **(B)** The infected phenotype of apple twigs inoculated with different strains at 5 dpi. **(C)** Lesion sizes produced by different strains on apple leaves at 3 dpi and apple twigs at 5 dpi. The mean lesion length was calculated from 7 apple leaves and 12 apple twigs. Bars represent the standard deviation. Asterisks indicate significant differences with the wild-type strain (*P* < 0.05).

To investigate whether four *VmGlu2* play role in the virulence, we performed pathogenicity assays of the wild-type strain and gene deletion mutants on detached apple leaves and twigs. Our results showed that *VmGlu2* deletion strains were significantly less virulent toward the apple leaves and twigs at 3 or 5 dpi, as compared to the wild-type stain, which typically displayed diseased symptoms of necrosis and canker (Figure 5). However, smaller lesions were found on the Δ*VmGlu2* inoculated apple leaves and twigs than those caused by the wild-type strain (Figures 5A,B). The Δ*VmGlu2* strains demonstrated a more than 60 and 76% reduction in the average lesion size on apple leaves at 3pi and twigs at 5 dpi. The complementation strain Δ*VmGlu2-C* recovered the high virulent phenotype that showed same symptoms on the apple leaves and twigs after reintroduction of *VmGlu2* into the Δ*VmGlu2* mutant (Figure 5C).

### Overexpression of *VmGlu2* Increased the Virulence of *V. mali*

**FIGURE 6:**
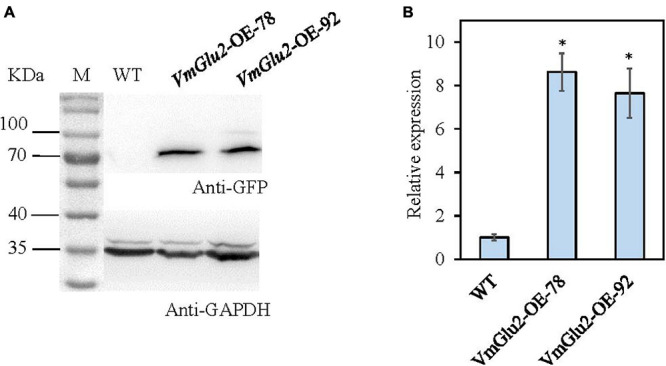
The verification of gene overexpression. **(A)** Western blot analysis of overexpressed VmGlu2 fused to the GFP-tag in the transformants grown in PDB (PDA without agar) for 2 days. The expression of GAPDH in *V. mali* was used as an endogenous control. **(B)** Expression level of *VmGlu2* in the *VmGlu2* overexpression (OE) transformants in apple twigs at 48 hpi. The expression level of *VmGlu2* in the apple bark tissues inoculation with the WT at 48 hpi was normalized to one. Asterisks represent significant differences (*P* < 0.05) in the expression level relative to that in the WT.

**FIGURE 7:**
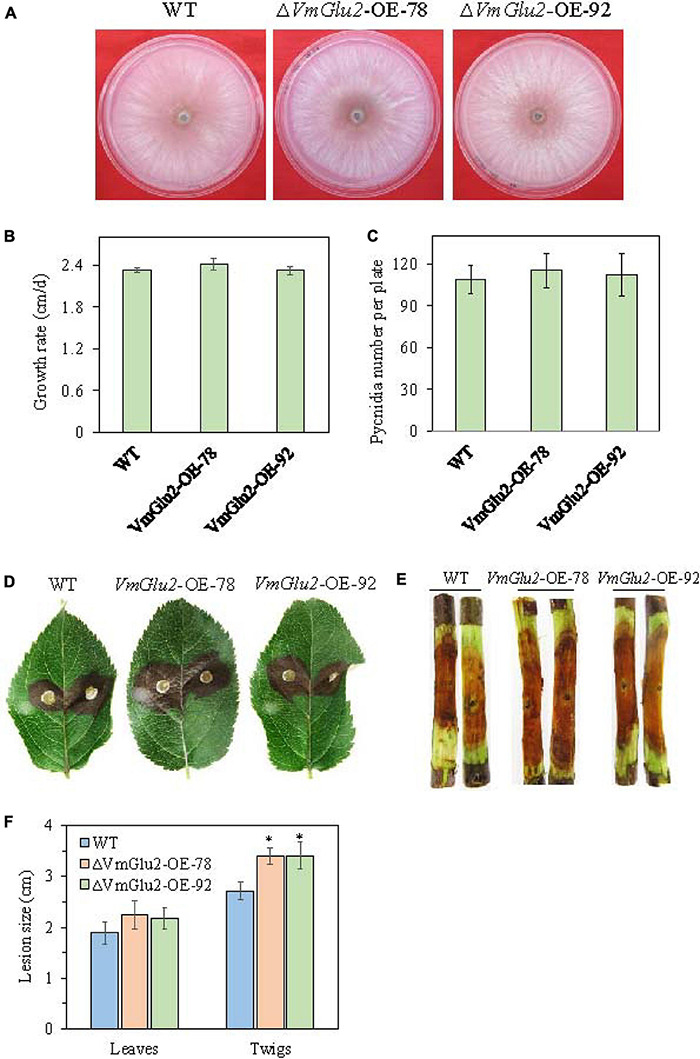
Overexpression of *VmGlu2* does not affect the growth and development of *V. mali*, but increases the virulence of *V. mali* in the leaves and twigs of *M. domestica* Borkh. cv. Fuji. **(A)** Colony morphology of WT and *VmGlu2* overexpression (OE) transformants for 4 days incubation on PDA. **(B)** Growth rate of WT and *VmGlu2* overexpression transformants on PDA for 3 days. **(C)** Number of pycnidia produced in the 9.0 cm per petri plate. Bars indicate standard deviation of means of four replicates. **(D)** Pathogenicity assay of the WT and *VmGlu2* overexpression (OE) transformants on apple leaves at 3 dpi. **(E)** Pathogenicity assay of WT and *VmGlu2* overexpression transformants on the apple twigs at 5 dpi. **(F)** Lesion sizes produced by different strains on the apple leaves at 3 dpi and apple twigs at 5 dpi. The mean lesion length was calculated from 6 apple leaves and 10 apple twigs. Bars represent the standard deviation. Asterisks indicate significant differences in OE transformants as compared to that in the WT (*P* < 0.05).

To further determine the function of *VmGlu2* on the pathogenicity of *V. mali*, the gene overexpression transformants were generated and detected by PCR (Supplementary Figure 4). The overexpression transformants were confirmed by Western blot analysis. When probed with Anti-GFP antibody, the wild-type strain showed no GFP protein signal was found, however, two transformants of *VmGlu2* overexpression (OE) displayed specific GFP detected bands with the expected size. Moreover, when probed with Anti-GAPDH antibody, all the strains exhibited specific immune signals (Figure 6A). Two *VmGlu2* overexpression transformants (*VmGlu2*-OE-78 and *VmGlu2*-OE-92) were selected for further analysis. Compared with the wild-type stain, the transcript levels of *VmGlu2* in *VmGlu2*-OE-78 and *VmGlu2*-OE-92 increased 8.6- and 7.6-fold, respectively, during the infection apple twigs (Figure 6B). Further analysis showed that overexpression of *VmGlu2* did not affect vegetative growth and pycnidia formation (Figures 7A–C). However, *VmGlu2* overexpression transformants significantly enhanced the virulence of *V. mali*. Larger lesions were observed on the *VmGlu2* overexpression transformants inoculated apple leaves (17% increase), especially apple twigs (25% increase) than those caused by the wild-type strain (Figures 7D–F). The phenotype and pathogenicity of the empty vector transformants and the wild-type strain were comparable (data not shown). These results verify that *VmGlu2* is a major virulence factor involved in the *V. mali* pathogenicity.

### Deletion and Overexpression of *VmGlu2* Affects β-Glucosidase Activity in *V. mali*

**FIGURE 8:**
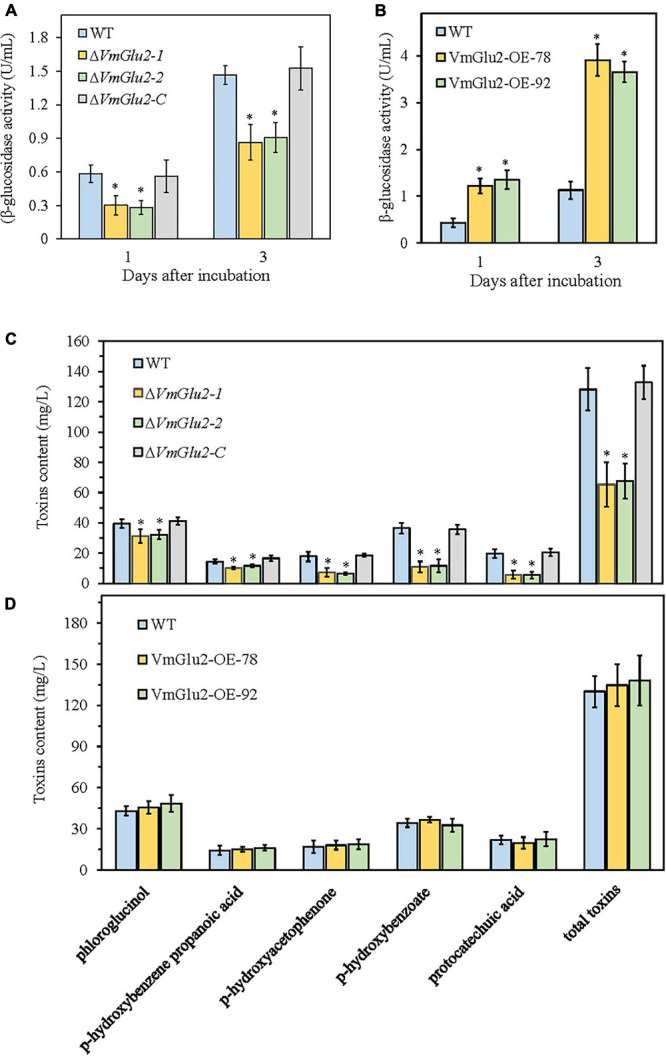
Effect of *VmGlu2* deletion and overexpression on the enzyme activity of β-glucosidase **(A,B)** and toxin production **(C,D)** in *V. mali*. The enzyme activity was measured in the apple branch extract media for different strains at 1 and 3 days after incubation. The pNPG was used as a substrate. Toxins contents were determined in the apple branch extract media for different strains after 7 days of growth. Bars indicate standard deviations of the mean of three replicates. Asterisks indicate significant differences in gene deletion mutants or OE transformants as compared to that in the wild-type strain (*P* < 0.05).

To detect VmGlu2 activity, the wild-type strain, gene deletion mutants, and overexpression transformants were cultured in the apple branch extract media. We determined β-glucosidase activity in the culture filtrates of all strains and uses pNPG as a substrate. The gene deletion strain Δ*VmGlu2* showed 50 and 39% reduction in the β-glucosidase activity at 1 and 3 dpi, respectively. The complementation strain Δ*VmGlu2-C* with the native gene restored β-glucosidase activity to the wild-type levels (Figure 8A). In contrast, the *VmGlu2* overexpression transformants exhibited an enhanced β-glucosidase activity, with 3.0- and 3.3-fold increases compared with the wild-type strain (Figure 8B). Thus, the results in enzyme activity assays suggest the deletion of *VmGlu2* significantly reduced the β-glucosidase activity, however, the gene overexpression enhanced the β-glucosidase activity.

### *VmGlu2* Deletion Affects Toxins Production by *V. mali*

To further reveal the mechanism underlying the virulence of *VmGlu2* deletion and overexpression, we examined the toxin levels in the apple branch extract media for different strains. All tested strains produced five kinds of toxins, however, the Δ*VmGlu2* mutants exhibited different degrees of reduction in the levels of five toxic compounds (Figure 8C). The *VmGlu2* deletion strains exhibited 20 (phloroglucinol) to 71% (protocatechuic acid) reduction in each toxic compounds than the wild-type strain. Moreover, the total toxins produced by the Δ*VmGlu2* mutants showed 48% decrease than the wild-type strain, whereas the complementation strain with the native gene restored the phenotype. In contrast, the toxin levels produced by the *VmGlu2* overexpression transformants were not significantly different than those produced by the wild-type strain (Figure 8D).

## Discussion

The role of CWDEs in the virulence of plant pathogens has been described by previous studies, while these enzymes are considered vital for the generation of disease symptoms and pathogenesis (Brito et al., 2006; Kubicek et al., 2014; Pérez-Hernández et al., 2017; Gui et al., 2018). The β-glucosidases are among the main CWDEs, and thus play important roles especially in the virulence of necrotrophic pathogens infection and disease development (Sasaki and Nagayama, 1996; Pérez-Hernández et al., 2017; Jourdan et al., 2018). *V. mali*, the causal agent of apple tree Valsa canker, infects host plants and causes typical symptoms through secreted CWDEs and toxins. Although previous studies have demonstrated that several CWDEs play crucial function in the pathogenicity of *V. mali*, the role of β-glucosidases in the pathogen virulence is still largely unknown. In the present study, we identified a β-glucosidase gene *VmGlu2* from *V. mali* that contains GH1 glycosyl hydrolase motif with β-glucosidase activities, while it is required for the full virulence of *V. mali*.

The bioinformatics analyses showed that VmGlu2 protein shared the characteristics of β-glucosidases and multiple conserved motifs (Brunner et al., 2002; de Giuseppe et al., 2014). The recombinant protein was successfully obtained by the prokaryotic expression system, while the purified VmGlu2 protein showed high activity (1.74 U/mL) of β-glucosidase using pNPG as a substrate at pH 5.5 and 50°, thus suggesting that the *VmGlu2* encoded a β-glucosidase and participated in the degradation of cellulose during the infection of *V. mali*. This result was in line with a previous study, which demonstrated that a 66 KDa protein of β-glucosidase from *F. oxysporum*, a phytopathogenic fungus, had high enzyme activities when using pNPG as a substrate at the similar reaction conditions (Alconada and Martínez, 1996).

Interestingly, in this study, the VmGlu2 protein could also catalyze the hydrolysis of phloridzin glucosides, and produce phloretin, which is the first step during toxin production in *V. mali* (Natsume et al., 1982; Gosch et al., 2010). Although the toxic compounds, leading to necrosis on apple tree, had long been identified, however, the specific functional genes involved in the degradation process of toxins in *V. mali* remain largely unknown. Previous study demonstrated that the candidate phloridzin-degrading enzyme gene *Vmlph1* had relationship with the vegetative growth. Moreover, it also participated in the virulence, conidiation and melanin biosynthesis in *V. mali*. Nevertheless, the deletion mutant of *Vmlph1* did not affect the ability of the pathogen to degrade phloridzin (Zhu et al., 2018). In addition, the function of Vmlph1 protein to degrade phloridzin still has not been verified. In this study, our results indicated that VmGlu2 had high enzymatic activities to hydrolyze phloridzin, thus suggesting that *VmGlu2* also plays crucial roles in toxins production by *V. mali*.

The β-glucosidases have been reported as pathogenicity factors in several plant pathogens. For example, the β-glucosidases involved in the virulence of *B. cinerea* and *S. scabies* (Sasaki and Nagayama, 1996; Jourdan et al., 2018), and disruption of the *BcSUN1* gene resulted in different cell surface alterations affecting the infection of *B. cinerea*, therefore decreasing its virulence potential (Pérez-Hernández et al., 2017). As in these examples from fungi and bacteria, *VmGlu2* is a critical pathogenicity factor in *V. mali*, as evidenced by its high expression during plant infection. Furthermore, we also found that *VmGlu2* exhibited higher transcript levels in the apple phloem than in the apple xylem. Based on the finding from previous studies that *V. mali* could grow rapidly and survived for a long time in the apple xylem, but it did not develop diseased symptoms, until the pathogen reached the phloem (Chen et al., 2016; Wang et al., 2018), we speculate that the expression pattern of *VmGlu2* is in line with their roles in the pathogenicity of *V. mali*.

Subsequently, we generated *VmGlu2* deletion and complementation stains to investigate the role of *VmGlu2* in the growth, development, and pathogenicity of *V. mali*. The Δ*VmGlu2* mutants exhibited similar morphology and growth rate of mycelia as compared to the wild-type strain. Intriguingly, our results revealed that *VmGlu2* was necessary for pycnidia formation and pathogenicity of *V. mali*. Moreover, the ability of producing pycnidia and pathogenicity was restored in the *VmGlu2* complementation stains. Similar results were demonstrated in other CWDEs in phytopathogens, for example, the deletion of cutinase *VdCUT11* did not affect mycelia growth and colony morphology, but it contributed to the virulence of *V. dahliae*, and the deletion mutants of feruloyl esterases *FAEs* exhibited a significant reduction in the pathogenicity. But it had no effect on the vegetative growth and development of *V. mali* (Gui et al., 2018; Xu et al., 2018). In addition, we obtained *VmGlu2* overexpression transformants, and found that the gene overexpression did not affect the vegetative growth and development, but it increased the pathogenicity of *V. mali*. Based on the deletion and overexpression of *VmGlu2* as well as pathogenicity assays, *VmGlu2* was confirmed to be involved in the virulence of *V. mali* in apple trees.

The conidia production is a vital phase in the life cycle of pathogens, therefore, it is generally believed that inhibition of pycnidia formation by *V. mali* could alleviate or effectively control the occurrence of apple tree Valsa canker. However, the relationship between conidiation and pathogenicity of *V. mali* is not yet clear. In a previous study, the deletion of *VmE02*, encoding the pathogen-associated molecular pattern (PAMP), demonstrated attenuated conidiation but not the virulence (Nie et al., 2019). Furthermore, the deletion of mitogen-activated protein kinase gene, *VmPmk1*, could lead to a reduced growth rate, and a decreased pathogen virulence. Meanwhile, it also leads to absence of pycnidia, thus implying no pycnidia production in mutants (Wu et al., 2017). The deletion mutant of *VmXyl1*, encoding the xylanase, produced normal growth rate and exhibited decreased pycnidia formation and virulence (Yu et al., 2018). Moreover, the deletion mutants of *VmVeA* and *VmVelB*, Velvet protein family members, showed increased conidiation and melanin production, but they exhibited reduced virulence (Wu et al., 2017, 2018; Yu et al., 2018). In the present study, the Δ*VmGlu2* mutants exhibited normal growth rate and decreased pycnidia formation and pathogenicity. These results suggest that conidiation and virulence of *V. mali* are not necessarily correlated.

The major pathogenic factors of *V. mali* involve multiple CWDEs and toxins (Natsume et al., 1982; Chen et al., 2012; Wang et al., 2014). Therefore, in this study, the VmGlu2 activities of β-glucosidase and production of toxins were investigated. The β-glucosidase activities of the Δ*VmGlu2* mutants were significantly lower, while the enzyme activities of the VmGlu2 overexpression transformants were notably higher, when compared with those of the wild-type strain, which was consistent with the reduced and enhanced virulence of the gene deletion and overexpression strains, respectively. Similarly, the deletion mutants of *VmXyl1* and most of the *FAEs* that exhibited significant decreases in virulence had significantly lower enzyme activities than the wild-type stains (Xu et al., 2018; Yu et al., 2018). Moreover, in *B. cinerea*, the deletion of *Xyn11A* resulted in a reduction in the xylanase activity and virulence, however, further study demonstrated that Xyn11A mainly contributed to the fungal virulence with its inducing necrosis of the infected plant tissue and not with its enzyme catalytic activity (Brito et al., 2006; Noda et al., 2010). In contrast, interruption of a xylanase gene, FGSG_03624, from *F. graminearum* demonstrated a significant reduction in the xylanase activity, but it did not impair its virulence (Sella et al., 2013). These complexities might be attributed to the functional redundancies of the CWDEs genes. By analysis the genome sequence of *V. mali*, we found three genes of GH1 family encode β-glucosidase with 40.7% identity. The three genes all have typical catalytic residues of GH1 enzymes in which two Glu act as the nucleophile and acid/base, respectively (de Giuseppe et al., 2014). In our previous study, we cloned *VmGlu1* (KY646110) and found the gene significantly up-regulated during *V. mali* infection (Li et al., 2017). However, the biological function of *VmGlu1* needs to be further studied.

In addition, our data also showed that toxin production by the Δ*VmGlu2* mutants were significantly lower than the wild-type strain, which also corresponds to the results of the reduced β-glucosidase activities and virulence. However, the overexpression of *VmGlu2* did not increase the toxin production. The degrading rates of phloridzin were both almost up to 100% for the wild-type and overexpression strains at 7 days after incubation in the apple branch extract media (data not shown). Therefore, we speculated that the β-glucosidases produced by *V. mali* were excessive to involve in the degradation of phloridzin. In apple and *E. amylovora* interaction, β-glucosidase activity was significantly higher in the susceptible genotype MM106 than that in the resistant genotype Evereste, which contributes to the fast multiplication of the bacteria in the leaves of MM106 (Gaucher et al., 2013). In the interaction of apple and *V. mali*, the phloridzin is converted into phloretin with the release of glucose by the action of VmGlu2. The phloretin is the precursor of five toxic compounds produced by *V. mali*, which determine the severity of necrosis on the apple tissues. On the other hand, the release of glucose could favor the growth of *V. mali* in apple tissues. Taken together, these data suggest that the role of β-glucosidases is complex in the virulence of *V. mali*. However, the functions of β-glucosidase genes and their cooperative roles deserve to be further investigated.

## Conclusion

We have demonstrated that the protein encoded by *VmGlu2* has high β-glucosidase activity when using the pNPG as a substrate, and it can convert phloridzin into phloretin while releasing the glucose. In addition, we reveal that *VmGlu2* is required for full pathogenicity of *V. mali* in apple tree, and it significantly influences its pycnidia formation. These results provide a vital evidence to explore further the complex functions of CWDEs in fungal pathogenicity.

## Data Availability Statement

Bioinformatics analysis data was downloaded from the National Center for Biotechnology Information (NCBI) database (URL: https://www.ncbi.nlm.nih.gov/) under the accession numbers TQN64568, KZL67576, RKL27771, RKL26803, AAL34084, BAA74958, AAF74209, BAA74959, QGZ11144, and AAM94393.

## Author Contributions

YH, CY, and CS carried out the experiments and analyzed the data with the help of MS. PL determined the toxins levels by HPLC. YH and CW wrote the manuscript with help from all authors. BL performed the manuscript revision and provided part of the financial support. MS reviewed and edited the manuscript. All authors approved its final version.

## Conflict of Interest

The authors declare that the research was conducted in the absence of any commercial or financial relationships that could be construed as a potential conflict of interest.

## Publisher’s Note

All claims expressed in this article are solely those of the authors and do not necessarily represent those of their affiliated organizations, or those of the publisher, the editors and the reviewers. Any product that may be evaluated in this article, or claim that may be made by its manufacturer, is not guaranteed or endorsed by the publisher.
