# Supplementary material for: β-Glucosidase VmGlu2 Contributes to the Virulence of Valsa mali in Apple Tree
Source: Front Microbiol. 2021 Jul 30;12:695112. doi: 10.3389/fmicb.2021.695112 (PMC8361449; doi:10.3389/fmicb.2021.695112)
Supplement: Supplementary file 1 [file Image_1.pdf]

Supporting Information Legends

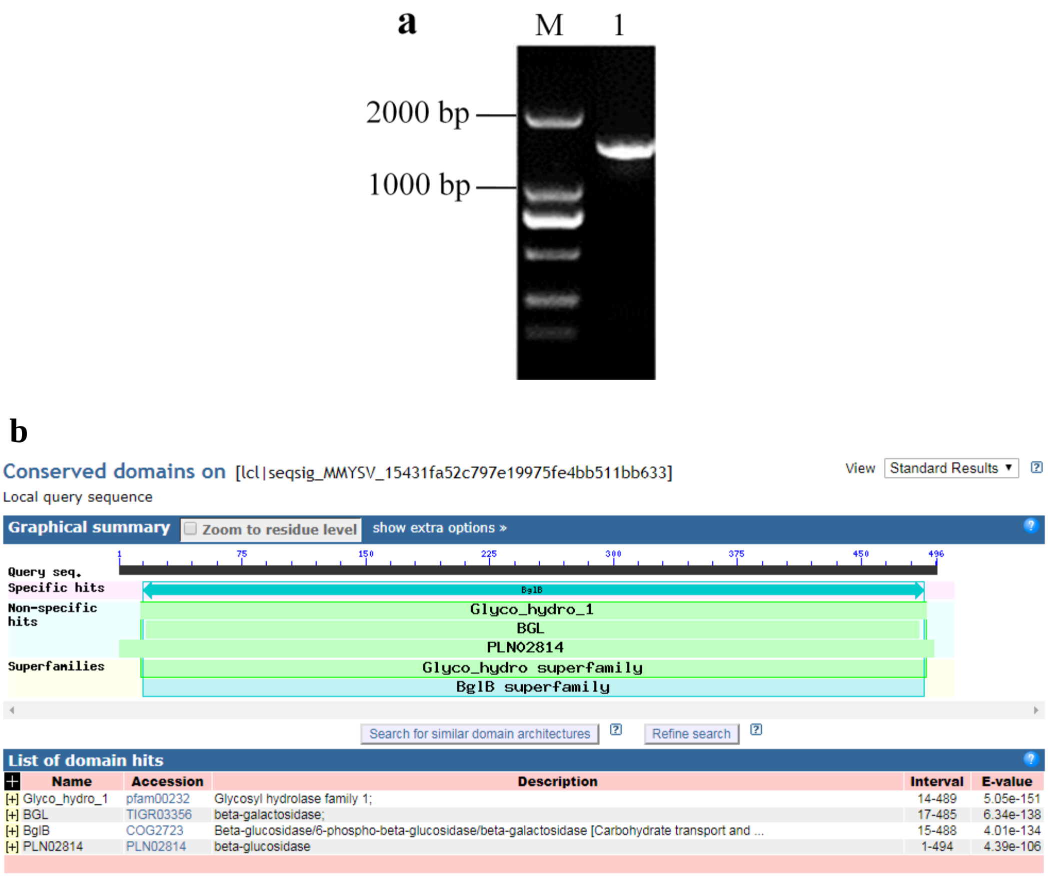

FIGURE S1 | RT-PCR amplification of the open reading frame (ORF) of *VmGlu2* (a) and conserved domains of VmGlu2 (b). The domain of VmGlu2 was predicted using NCBI's conserved domain database.

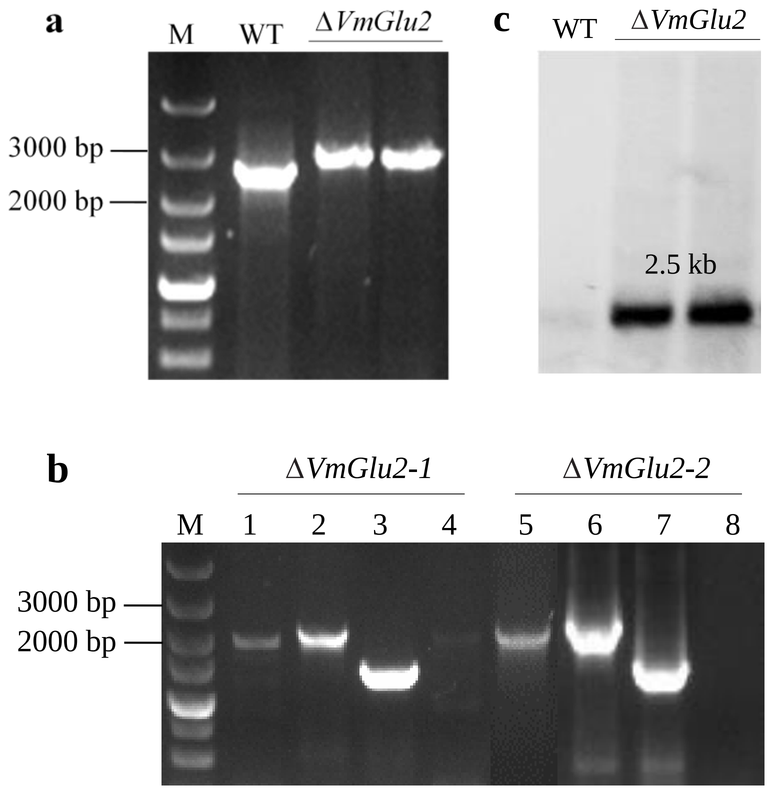

FIGURE S2 | Gene deletion verification of *VmGlu2* by PCR and Southern blot analysis. (a) Detection of *VmGlu2* detection mutants by PCR with detection primer pairs with primer pair *VmGlu2*-I-F/R (detection of fusion segment). (b) Detection of *VmGlu2* detection mutants by PCR with four primer pairs. Lane 1 and 5, *VmGlu2*-Up-F/HPH-R (detection of upstream of *VmGlu2* and *HPH* gene); Lane 2 and 6, HPH-F/*VmGlu2*-Down-R (detection of HPH gene and downstream of *VmGlu2*); Lane 3 and 7, HPH-F/HPH-R (detection of HPH resistance gene); Lane 4 and 8, *VmGlu2*-F/*VmGlu2*-R (detection of *VmGlu*2 gene). (c) Further confirmation of *VmGlu2* gene deletion mutants by Southern blot.

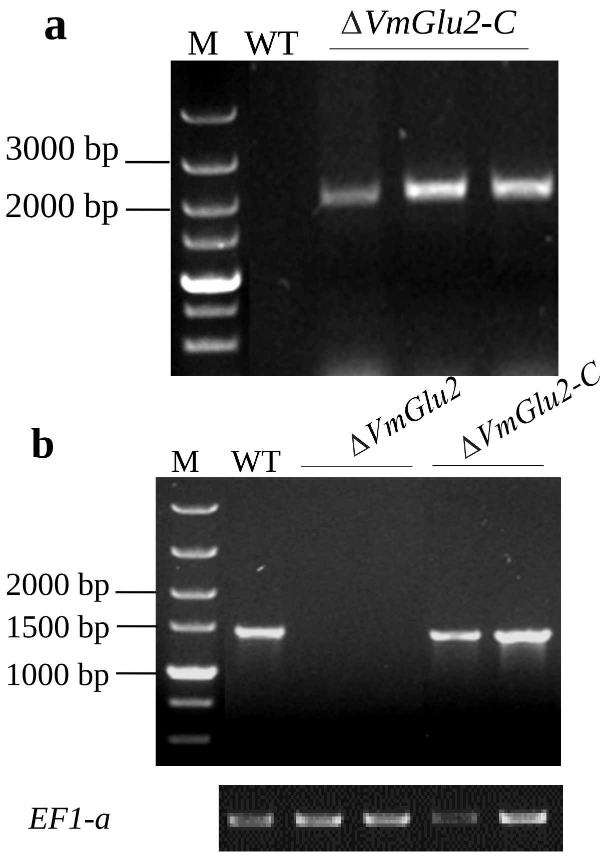

FIGURE S3 | Detection of complementation strains by PCR and RT-PCR. (a) The primer pair *VmGlu2*-ID-F/R was used to detect the complementary fragments by PCR from the genomic DNA of *VmGlu2* gene complementation strains. (b) Detection of *VmGlu2* from the wild-type strain, *VmGlu2* deletion mutants and complementation strains by RT-PCR. The housekeeping gene *EF1-a* was used as control.

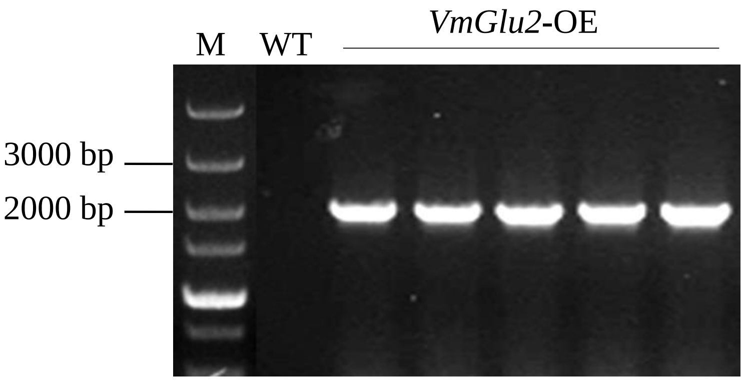

FIGURE S4 | Detection of gene overexpression (OE) transformants by PCR. The primer pair *VmGlu2*-ID-F/R was used to detect *VmGlu2* overexpression transformants.
