# Supplementary material for: β-Glucosidase VmGlu2 Contributes to the Virulence of Valsa mali in Apple Tree
Source: Front Microbiol. 2021 Jul 30;12:695112. doi: 10.3389/fmicb.2021.695112 (PMC8361449; doi:10.3389/fmicb.2021.695112)
Supplement: Supplementary file 2 [file Table_1.docx]

**Table S1 Primers used in this study**

| **Fragments Use** | **Primers** | **Sequence (5'-3')** |  |
| --- | --- | --- | --- |
| Amplification ORF | *VmGlu2*-F | ATGATGTACTCAGTCTCTG | |
|  | *VmGlu2*-R | TTACTTCCGGATAAGATG | |
| Prokaryotic expression | *VmGlu2*-YH-F | CCGGAATTCATGATGTACTCAGTCTCTG | |
|  | *VmGlu2*-YH-R | ATAGTTTAGCGGCCGCTTACTTCCGGATAAGATG | |
| qRT-PCR | *EF1-α*-F | TGAGTTCGAGGCTGGTATCTCCAA | |
|  | *EF1*-*α*-R | TGTCCATCTTGTTGATGGCGACGA | |
|  | qRT-*VmGlu2*-F | AGGACGACCCCATTAACGAGA | |
|  | qRT-*VmGlu2*-R | TGCTTCGAGCGATTCGGTGAC | |
| Deletion of *VmGlu2* | *VmGlu2*-Up-F | CTGTCGAACGACAATCAATGG | |
|  | *VmGlu2*-Up-R | caaaataggcattgatgtgttgacctccTGTCTGATCGGCCAGCATTAT | |
|  | *VmGlu2*-Down-F | ctcgtccgagggcaaaggaatagagtagACGTAGTCTATAGCAGAGGAA | |
|  | *VmGlu2*-Down-R | ATGACGTACCATCCTCTTTGA | |
|  | *VmGlu2*-nest-F | GTTGCATACTCTGCATGCTAA | |
|  | *VmGlu2*-nest-R | ACCATAAATGTTGAGACCAGG | |
|  | *VmGlu2*-I-F | AGTCGGCTGCTTTTATACCTC | |
|  | *VmGlu2*-I-R | ACAGCATCACCATCAAAGTCA | |
|  | HPH-F | GGAGGTCAACACATCAATGCC | |
|  | HPH-R | CTACTCTATTCCTTTGCCCTCGG | |
| Complementary of *VmGlu2* | *VmGlu2*-Native-F | actcactatagggcgaattgggtactcaaattggttGGCCAAAATGCTGTCTGTTGC | |
|  | *VmGlu2*-Native-R | caccaccccggtgaacagctcctcgcccttgctcacCCATCAAAGTCATCTATCACA | |
|  | *VmGlu2*-ID-F | ATGATGTACTCAGTCTCTG | |
|  | *VmGlu2*-ID-R | GACACGCTGAACTTGTGGCCGTT | |
| Overexpression of *VmGlu2* | VmGlu2-OE-F | tttcgtaggaacccaatcttcaaaATGATGTACTCAGTCTCTGATTTCGAC | |
|  | VmGlu2-OE-R | caccaccccggtgaacagctcctcgcccttgctcacTTACTTCCGGATAAGATGCC | |
